# Longitudinal Associations Between COVID-19 Stress and the Mental Health of Children With ADHD

**DOI:** 10.1177/10870547231168334

**Published:** 2023-04-25

**Authors:** Ainsley Summerton, Susannah T. Bellows, Elizabeth M. Westrupp, Mark A. Stokes, David Coghill, Mark A. Bellgrove, Delyse Hutchinson, Stephen P. Becker, Glenn Melvin, Jon Quach, Daryl Efron, Argyris Stringaris, Christel M. Middeldorp, Tobias Banaschewski, Emma Sciberras

**Affiliations:** 1 Deakin University, Geelong, VIC, Australia; 2 La Trobe University, Bundoora, VIC, Australia; 3 University of Melbourne, Parkville, VIC, Australia; 4 Murdoch Children’s Research Institute, Parkville, VIC, Australia; 5 The Royal Children’s Hospital, Parkville, VIC, Australia; 6 Monash University, Clayton, VIC, Australia; 7 University of New South Wales, Sydney, NSW, Australia; 8 Cincinnati Children’s Hospital Medical Center, Cincinnati, OH, USA; 9 University of Cincinnati College of Medicine, OH, USA; 10 University College London, UK; 11 National and Kapodistrian University of Athens, Greece; 12 University of Queensland, Brisbane, QLD, Australia; 13 Children’s Health Queensland Hospital and Health Service, Brisbane, QLD, Australia; 14 Heidelberg University, Germany

**Keywords:** ADHD, COVID-19, stress, longitudinal, mental health

## Abstract

**Objective:**

To investigate the longitudinal associations between COVID-19 induced stress (related to COVID-19 restrictions/changes), attention-deficit/hyperactivity disorder (ADHD) symptoms, oppositional symptoms, and mental health outcomes (negative affect, anxiety, depression, and irritability) in children with ADHD during the COVID-19 pandemic.

**Method:**

Parents of 140 Australian children with ADHD (aged 5–17 years) completed an online survey in May 2020 during stay-at-home restrictions and 12-months later.

**Results:**

Baseline COVID-19 stress was associated with increased total ADHD symptom severity (β = .21, *p* = .007) and hyperactivity/impulsivity symptoms (β = .23, *p* = .002) at 12-months, after accounting for covariates (i.e., child age, gender, ADHD medication, socio-economic status, and baseline symptoms). Despite some indication of associations between baseline COVID-19 stress and 12-month oppositional symptoms and negative affect, these were attenuated when adjusting for baseline symptoms.

**Conclusions:**

The study provides initial evidence of the medium-term impacts of pandemic-related stress for children with ADHD.

Prior to the COVID-19 pandemic, studies have reported that children with attention-deficit/hyperactivity disorder (ADHD) experience greater overall perceived stress compared to children without ADHD (Frick et al., 2022), and that the experience of stress during childhood and adolescence can have a prolonged influence on future ADHD symptom severity (Hartman et al., 2019; Humphreys et al., 2019). Children with ADHD may be more sensitive to the effects of stress compared to their peers without neurodevelopmental conditions (Hartman et al., 2019). Experiencing multiple stressors over time may have an additive effect on ADHD symptom severity and other mental health symptoms (Rydell, 2010). For example, exposure to stressors in childhood such as illness, conflict, and school pressure have been associated with increased ADHD symptom severity in adolescence (Humphreys et al., 2019). High levels of stress exposure have also been prospectively associated with persistent ADHD symptoms and emotion dysregulation from childhood into young adulthood (Hartman et al., 2019).

The COVID-19 pandemic is a recent and chronic global health emergency and stressor that may be impacting negatively on the adjustment of children with ADHD. The first COVID-19 case in Australia was recorded in January 2020 and led to a nationwide lockdown from March to May 2020 (Department of Health, 2020). As was the case in many countries, this lockdown resulted in adults working from home, children participating in remote learning, and reliance on the internet for social interactions and connecting with friends and family (Sciberras et al., 2022). Ongoing lockdowns, social distancing measures, and travel restrictions were present throughout Australia, with the State of Victoria entering a second strict lockdown in July to October 2020, which comprised of a night-time curfew, a 5-km (3.1 mile) travel radius, and limited reasons to leave home. Other parts of Australia experienced multiple, rapidly enforced lockdowns (e.g., 3 days to 2 weeks) to contain the virus. For example, the State of Victoria entered a fourth lockdown in May 2021 for 2 weeks and a fifth in July 2021 for 12 days due to several new cases (24–26) identified in the community (State Government of Victoria, 2021).

The pandemic has been a major life stressor in Australia and internationally, including fear of contracting the COVID-19 virus, as well as stress potentially arising from government enforced social distancing restrictions, significant lifestyle constraints, and changes to social connections with family and friends. Pandemic-related stressors such as these have led to a 25% increase in anxiety and depression worldwide, according to a report by the World Health Organization (2022). An Australian survey found that 38% of adults had experienced at least one COVID-19 related stressor in October 2020 and that 23% were still experiencing COVID-19 related stress in April 2021 despite some relaxation of restrictions (Australian Bureau of Statistics [ABS], 2021).

The uncertainties and changes that have arisen as a result of the pandemic have also been stressful for many children, and this stress may be acted out through increased challenging behaviors (Panda et al., 2021) or anxiety (Imran et al., 2020). Increased rates of mental health problems in children during the pandemic have also been reported, with one meta-analysis finding that 70% to 90% of children experienced a decline in mental health during lockdown (Panda et al., 2021). More specifically, studies have found elevated child stress, anxiety, and depressive symptoms (Panda et al., 2021), irritability and inattention (Cost et al., 2022; Panda et al., 2021), hyperactivity (Cost et al., 2022), and oppositional symptoms (Imran et al., 2020). Additionally, one study found associations between child stress during lockdown, and elevated irritability, inattention, and hyperactivity (Cost et al., 2022). Given that children with ADHD may be particularly impacted by stress, understanding the consequences that COVID-19 induced stress may have had on ADHD symptoms and broader mental health symptoms is essential.

Studies specifically focusing on children with ADHD during the pandemic have reported increased ADHD symptoms (Zhang et al., 2020), irritability (Shah et al., 2021), and oppositional symptoms (Breaux et al., 2021). Further, elevated ADHD symptom severity during the pandemic has been associated with home-learning difficulties (Becker et al., 2020; Sciberras et al., 2022), boredom (Melegari et al., 2021; Shah et al., 2021; Sibley et al., 2021), and trauma reactions to lockdown, mediated by irritability (Uçar et al., 2022). Increased symptoms of anxiety (Shah et al., 2021) and depression (Breaux et al., 2021) in children with ADHD have also been evident in the early stages of the pandemic. However, the role of stress in understanding the observed increase in ADHD symptom severity and mental health symptoms during the pandemic has been limited.

A cross-sectional study by Sciberras et al. (2022) investigated the impacts of the pandemic in a sample of 213 children aged 5 to 17 years with ADHD using parent report. The study found that approximately one-third of children with ADHD experienced moderate to extreme COVID-19 stress in the early stages of the pandemic, which was independently associated with poorer mental health outcomes including worry, sadness, anxiety, fatigue, loneliness, irritability, distractibility, and fidgeting, and occurred irrespective of ADHD medication use and pre-existing co-occurring internalizing and externalizing conditions (Sciberras et al., 2022). However, the cross-sectional design limits capacity to understand whether COVID-19 stress predicts symptom severity, and less is known about the longitudinal relationships between stress, ADHD symptoms, and mental health symptoms as the pandemic continues. Additionally, mental health symptoms were assessed using single items, rather than a more comprehensive measure of each mental health domain. For example, single items assessed distractibility and fidgeting rather than a validated ADHD rating scale.

The current study examined the same cohort with longitudinal data to investigate whether COVID-19 stress experienced in the early stages of the pandemic was prospectively associated with ADHD symptom severity, oppositional symptoms, and domains of mental health including negative affect, anxiety, depression, and irritability 12-months later. It was hypothesized that increased levels of COVID-19 stress at baseline would be associated with greater frequency and severity of ADHD, oppositional, and mental health symptoms 12-months later, after accounting for child age, gender, ADHD medication use, neighborhood socio-economic status, and baseline symptoms.

## Method

### Study Design

This study used two waves of data (baseline and 12-month follow-up) from the ADHD COVID-19 study, which is a longitudinal study surveying parents of Australian children with ADHD during the COVID-19 pandemic. The study was approved by the Deakin University Health Faculty Ethics Committee (HEAG-H 60_2020).

### Participants and Procedure

Participants were recruited in May 2020 through ADHD organizations, social media, and ADHD parent support groups. Parents and primary caregivers (>18 years old) living in Australia with a child aged 5 to 17 years who had been diagnosed with ADHD were eligible to participate. Of 221 participants recruited at baseline, 207 remained in the study and were invited by email to take part in a 12-month follow-up survey in May 2021. The survey was completed online through Research Electronic Data Capture (REDCap; Harris et al., 2009, 2019). The 12-month follow-up survey was open for 8 weeks. Weekly reminders for the 12-month follow-up were sent to participants using a range of communication methods including email, text message, and two phone call attempts, with a final reminder email sent 1 week before the survey closed. Participants were given a $20 e-voucher after completing the 12-month survey to thank them for their time.

### Materials and Measures

#### Child COVID-19 Stress

The CoRonavIruS Health and Impact Survey (CRISIS; Nikolaidis et al., 2021) measures the impact of pandemic-induced life changes on mental health. The following four items from the Life Changes domain of the CRISIS were used to create a COVID-19 stress measure in the baseline survey for parent-report: (1) How stressful have the restrictions on leaving home been for your child? (2) How stressful have these changes in family contacts been for your child? (3) How stressful have these changes in social contacts been for your child? (4) How much has cancellation of important events in your child’s life been difficult for him/her? Items were scored on a 5-point Likert scale (1 = *not at all* to 5 = *very extremely*), where higher scores indicated greater stress. The Life Changes domain of the CRISIS has demonstrated good internal reliability (ω = .73–.77) and high consistency between US and UK samples (*r* = .91–.99) using parent report (Nikolaidis et al., 2021). The COVID-19 stress measure developed specifically in this study has also been validated through a confirmatory model (Sciberras et al., 2022). Cronbach’s alpha for the sample used in the current study showed an acceptable level of internal reliability for the COVID-19 stress measure, α = .79.

#### ADHD Symptom Severity and Oppositional Symptoms

ADHD and oppositional symptoms were measured at baseline and 12 months using the 26 item Swanson, Nolan, and Pelham Rating Scale—Fourth Version (SNAP-IV; Swanson et al., 2001). Parents reported on their child’s inattention (nine items), hyperactivity/impulsivity (nine items), and oppositional symptoms (eight items) on a 4-point Likert scale (0 = *not at all* to 3 = *very much*), with higher scores indicating increased symptom severity. The inattention and hyperactivity/impulsivity subscales were also combined to provide an ADHD symptom severity total score. The SNAP-IV has previously demonstrated excellent internal reliability for parent ratings (α = .94; Bussing et al., 2008), and in the current sample, was excellent for ADHD symptom severity (α = .91) and inattention symptoms (α = .90), and very good for hyperactivity/impulsivity symptoms (α = .88) and oppositional symptoms (α = .89) at the 12 month time point.

#### Mental Health

##### Negative Affect

Ten items from the Mood States domain of the CRISIS were used at baseline and 12 months for parents to report their child’s mood from the circumplex model of affect, including worry, happy versus sad, enjoying usual activities, anxiety, fatigue, irritability, fidgety or restless, difficulty to concentrate or focus, loneliness, and negative thoughts. Scores were measured on a 5-point Likert Scale for each mood state (1 = *not at all* to 5 = *extremely*) except for happy versus sad and enjoying activities, which were reverse scored. A total score was calculated, with higher scores indicating more negative mood states. Cronbach’s alpha showed very good internal reliability within the sample at 12 months, α = .82.

##### Depression

Parents reported on their child’s depressive symptoms using the Short Mood and Feelings Questionnaire (SMFQ) at baseline and 12 months. The SMFQ is a reliable 13-item measure (α = .85–.87; Angold et al., 1995) assessing depressive symptoms using a 3-point Likert Scale (0 = *not true* to 2 = *true*). Higher scores indicated greater symptoms of depression. The SMFQ showed excellent internal reliability within the sample at 12 months, α = .90.

##### Anxiety

The brief version of the Spence Children’s Anxiety Scale (SCAS; Spence, 1997) was used to assess parent-reported anxiety at baseline and 12 months. The brief 8-item version (Spence et al., 2014) measures anxiety on a 4-point Likert scale (0 = *never* to 3 = *always*), with higher scores indicating greater symptoms of anxiety. The original SCAS has shown excellent reliability (α = .92; Spence et al., 2003) and has been validated for use by parents of children aged 6 to 18 years (α = .89; Nauta et al., 2004). Cronbach’s alpha showed a very good level of internal reliability within the sample at 12 months, α = .85.

##### Irritability

The Affective Reactivity Index (ARI) was used at baseline and 12 months to measure parent-reported chronic irritability symptoms on seven items using a 3-point Likert Scale (0 = *not true* to 2 = *certainly true*). Higher scores indicated greater irritability. The ARI has been validated for parent-report in a clinical sample longitudinally, including a percentage of children with ADHD (α = .89–.92; Stringaris et al., 2012). The ARI demonstrated excellent internal reliability within the sample at 12 months, α = .91.

#### Demographics

In the baseline survey, parents reported their country of birth, Australian state of residence (by postcode), primary language spoken at home, Aboriginal or Torres Strait Islander status, high school completion, number of children in the home, child age, and child gender. Information regarding the child’s ADHD medication use, and diagnosis or treatment of any co-occurring internalizing (anxiety and depression) or externalizing (oppositional defiant disorder and conduct disorder) conditions were assessed at baseline and the 12-month follow-up. The Socio-Economic Indexes for Areas (SEIFA; ABS, 2018) was calculated as a general socio-economic indicator based on participants’ postcode at baseline.

### Statistical Analyses

To be included in the analyses, participants were required to have data available on COVID-19 stress at baseline, and at least one outcome of interest at the 12-month follow-up. Independent samples *t*-tests and chi-square tests for independence examined any differences in child age, neighborhood socio-economic status, child gender, and state of residence between those included and excluded from the analyses. Descriptive statistics were used to summarize the demographic and individual characteristics of the sample, as well as the range and average responses for the outcomes of interest.

Linear regression analyses examined the associations between baseline COVID-19 stress (independent variable) and 12-month ADHD symptom severity (including inattention and hyperactivity/impulsivity subscales), oppositional symptoms, negative affect, anxiety, depression, and irritability (dependent variables) at 12 months. Two sets of adjusted analyses for each domain were also undertaken. First, adjusted linear regression analyses were conducted to account for covariates—ADHD medication use, child age, gender, and neighborhood socio-economic status. Second, adjusted analyses were re-run to account for baseline symptoms of the dependent variable of interest and covariates. Due to the number of statistical comparisons, a False Discovery Rate (FDR) correction was used for both sets of adjusted analyses using *q* = 0.0125 as the threshold for significance (Benjamini & Hochberg, 1995). IBM SPSS Statistics (Version 28) was used to complete all data analyses.

## Results

### Sample Characteristics

Seventy percent of invited participants completed the 12-month survey (see Supplemental Figure 1); with 140 responses eligible for analysis. There was no significant difference between the age of children included in the analyses (*n* = 140, *M* = 10.59 years, *SD* = 3.00) and excluded (*n* = 65, *M* = 10.62 years, *SD* = 3.20), *t*(203) = 0.05, *p* = .961, 95% confidence interval (CI) [−0.89, 0.93], or neighborhood socio-economic status between those included (*n* = 136, M = 1,040.85, *SD* = 52.36) and excluded (*n* = 67, M = 1,033.25, *SD* = 46.89), *t*(201) = −1.01, *p* = .316, 95% CI [−22.49, 7.31]. There was no significant effect of gender distribution χ^2^ (*df* = 1, *n* = 204) = 0.14, *p* = .706, θ = −0.03, or state of residence χ^2^ (*df* = 6, *n* = 207) = 5.47, *p* = .485, θ = 0.16, between those included and excluded.

As shown in Table 1, the average age of the sample at baseline was 10.6 years and most children were male. Co-occurring internalizing conditions were more prevalent than externalizing conditions. There were very similar rates of ADHD medication use at baseline (85%) and 12 months (87%). Most parents were female and had completed high school. Approximately 60% of the sample lived in New South Wales or Victoria. The average scores for each outcome of interest remained similar across the two time points (see Supplemental Table 1).

**Table 1.** Demographic Characteristics of the Sample.

Characteristics	*n* (*M*, (*SD*))	% (range)
Primary caregiver characteristics		
Born in Australia^a^	110	80.9
Aboriginal or Torres Strait Islander	4	2.9
Primary caregiver female	137	97.9
State of residence		
Australian Capital Territory	9	6.4
New South Wales	41	29.3
Victoria	42	30.0
Queensland	16	11.4
South Australia	1	0.7
Western Australia	30	21.4
Tasmania	1	0.7
High school completion	125	89.3
Family characteristics		
English spoken at home	136	97.1
Number of children at home, *M* (*SD*), range	2.2 (0.8)	1.0–5.0
SEIFA^a,b^, *M* (*SD*), range	1,040.85 (52.36)	879–1,128
Child characteristics		
Child age at baseline, *M* (*SD*), range	10.6 (3.0)	6.0–17.0
Gender (Male)	106	75.7
Primary school age^c^	76	61.3
Co-occurring condition at baseline or 12 months^d^		
Internalizing	98	70.5
Externalizing	29	20.9
ADHD medication use		
Baseline^d^	109	85.2
12 months^d^	111	86.7
Baseline and/or 12 months^e^	127	91.4

a Missing = 4.

b Socio-economic indexes for areas.

c Missing = 16.

d Missing = 1.

e Missing = 12.

### Longitudinal Associations Between COVID-19 Stress and Outcomes at 12 Months

Bivariate correlations between the variables of interest can be found in Supplemental Table 2. In the unadjusted models, baseline COVID-19 stress was significantly associated with increased 12-month ADHD symptom severity (β = .22, *p* = .009), and more specifically, increased hyperactivity/impulsivity symptoms (β = .25, *p* = .003; see Table 2). COVID-19 stress was also significantly associated with increased oppositional symptoms (β = .19, *p* = .029), and negative affect (β = .20, *p* = .020) at 12 months (see Table 2). There was no significant association between COVID-19 stress and inattention symptoms, anxiety, depression, or irritability.

**Table 2.** Unadjusted Linear Regression Analyses Between Baseline COVID-19 Stress and Symptoms at 12 Months.

	Unadjusted COVID-19 related stress^a^		
	*B* [95% CI]	β	*p*-Value
ADHD symptom severity	2.49 [0.64, 4.33]	.22	.009
Inattention	0.81 [−0.19, 1.80]	.14	.110
Hyperactivity/impulsivity	1.68 [0.57, 2.79]	.25	.003
Opposition	1.14 [0.12, 2.17]	.19	.029
Negative affect	0.14 [0.02, 0.25]	.20	.020
Anxiety	0.56 [−0.36, 1.48]	.10	.233
Depression	0.55 [−0.55, 1.65]	.08	.325
Irritability	0.55 [−0.10, 1.19]	.14	.094

a *N* = 138–140.

When adjusting for covariates, baseline COVID-19 stress remained significantly associated with increased ADHD symptom severity (β = .26, *p* = .001) and hyperactivity/impulsivity symptoms (β = .29, *p* < .001) at 12 months. While associations were also shown between COVID-19 stress and increased oppositional symptoms (β = .18, *p* = .034), and negative affect (β = .20, *p* = .024), these findings did not survive FDR correction (see Table 3). When adjusting for covariates and baseline symptoms, baseline COVID-19 stress remained associated with increased ADHD symptom severity (β = .21, *p* = .007) and hyperactivity/impulsivity symptoms (β = .23, *p* = .002) at 12 months (see Table 3). Both associations survived FDR correction. An expanded version of this table including baseline symptoms and covariates is presented in Supplemental Table 3.

**Table 3.** Adjusted Linear Regression Analyses Between Baseline COVID-19 Stress and Symptoms at 12 Months.

	Adjusted model 1—COVID-19 stress with covariates^a,b^				Adjusted model 2—COVID-19 stress with covariates and baseline symptoms^c,d^			
	*B* [95% CI]	β	*p*-Value	*sr* ^2^	*B* [95% CI]	β	*p*-Value	*sr* ^2^
ADHD symptom severity	2.91 [1.15, 4.68]	.26	.001*	.068	2.43 [0.67, 4.19]	.21	.007*	.042
Inattention	0.98 [−0.02, 1.99]	.17	.055	.027	0.82 [−0.20, 1.84]	.13	.113	.017
Hyperactivity/impulsivity	1.93 [0.91, 3.00]	.29	<.001*	.082	1.59 [0.62, 2.55]	.23	.002*	.050
Opposition	1.14 [0.09, 2.18]	.18	.034	.033	0.67 [−0.34, 1.68]	.10	.191	.010
Negative affect	0.14 [0.02, 0.26]	.20	.024	.039	−0.05 [−0.19, 0.09]	−.07	.468	.004
Anxiety	0.39 [−0.57, 1.34]	.07	.422	.005	0.16 [−0.74, 1.06]	.03	.722	.001
Depression	0.52 [−0.62, 1.65]	.08	.371	.006	−0.35 [−1.51, 0.80]	−.05	.547	.003
Irritability	0.56 [−0.11, 1.23]	.14	.101	.020	0.11 [−0.56, 0.78]	.03	.745	.001

a *N* = 133–135.

b Adjusted for ADHD medication use, child age, gender, and neighborhood socio-economic status (SEIFA).

c *N* = 116–119.

d Adjusted for baseline symptoms, ADHD medication use, child age, gender, and SEIFA.

* *p*-Values below FDR threshold of *p* = .0125.

## Discussion

This study aimed to investigate the longitudinal associations between children’s COVID-19 stress and ADHD symptom severity, oppositional symptoms, negative affect, anxiety, depression, and irritability in Australian children aged 5 to 17 years with ADHD at two-time points during the pandemic. The hypothesis was partially supported, as COVID-19 stress reported in the early months of the pandemic was significantly associated with greater ADHD symptom severity, hyperactivity/impulsivity symptoms, oppositional symptoms, and increased negative affect 12 months later after accounting for covariates. COVID-19 stress also continued to be associated with greater ADHD symptom severity, specifically hyperactivity/impulsivity symptoms when accounting for baseline symptoms.

The significant association shown between baseline COVID-19 stress and 12-month ADHD symptom severity is consistent with pre-pandemic research indicating associations between stress and exacerbated ADHD symptom severity across time in childhood and adolescence (Hartman et al., 2019; Humphreys et al., 2019). The results also support the cross-sectional associations in the early months of the pandemic between COVID-19 stress and symptoms of ADHD such as fidgeting (Sciberras et al., 2022). The current study expands these findings by indicating ongoing associations between COVID-19 stress and ADHD symptom severity over 12 months, independent of the influence of demographic variables, medication use, and baseline symptoms in the early months of the pandemic.

However, there were differences between specific ADHD symptoms. While baseline COVID-19 stress was associated with symptoms of hyperactivity/impulsivity at 12 months, there was no significant association with inattention symptoms, despite previous research indicating a deterioration in focus and attention in children with ADHD during the early stages of the pandemic (Breaux et al., 2021; Sciberras et al., 2022; Zhang et al., 2020). These findings support claims made by Imran et al. (2020) that pandemic-related stress in children may be more likely to manifest as externalizing symptoms. During stay-at-home restrictions, children have spent more time indoors and had less opportunity to socialize with friends. Additionally, children with ADHD have experienced increased feelings of boredom (Melegari et al., 2021; Shah et al., 2021; Sibley et al., 2021), restlessness, and lack of interest in activities (Melegari et al., 2021) during the pandemic. As boredom has been linked to distress (Shah et al., 2021) and ADHD symptom severity (Melegari et al., 2021), it is possible that externalizing symptoms may be more susceptible to stress than other mental health symptoms. This may explain the associations shown between COVID-19 stress and hyperactivity/impulsivity symptoms and to an extent, oppositional symptoms. However, the association between baseline COVID-19 stress and 12-month oppositional symptoms should be interpreted with caution as this association did not survive FDR correction when accounting for covariates. Supports that are targeted and specific to addressing the symptoms most strongly impacted by stress could be beneficial to improve outcomes for children with ADHD and their families as the pandemic continues.

There was also some evidence of a prospective association between COVID-19 stress in the early months of the pandemic and greater negative affect 12 months later, albeit not in other domains of mental health such as anxiety and depression. Again, however, this association did not hold after FDR correction for multiple comparisons, so should be interpreted with caution and requires further research. These findings were unexpected given the associations identified between stress and aspects of mental health such as anxiety, depression, and irritability for children with ADHD prior to (Hartman et al., 2019; Humphreys et al., 2019), and during (Sciberras et al., 2022; Shah et al., 2021), the pandemic. One explanation for the non-significant associations in the current study may be the reported benefits for children with ADHD in the early stages of the pandemic such as consistent routine (Melegari et al., 2021) or more time spent with family (Sibley et al., 2021). Such benefits have been associated with decreased mental health symptoms during the pandemic (Dvorsky et al., 2022).

There are several clinical implications stemming from this study. For clinicians working with children with ADHD, it is important to enquire about, and acknowledge, the various causes of stress relating to the pandemic and the impacts this may have on children’s symptoms. While general clinical guidance has suggested that children with ADHD should continue to be supported in accessing and monitoring medication use during the pandemic (Cortese et al., 2020), they may also require additional assistance to foster effective and positive coping strategies during the pandemic (Saline, 2021). For example, one study found that consistent routine is especially important for children with ADHD to reduce stress during the pandemic (Dvorsky et al., 2022). In the early stages of the pandemic, many children with ADHD were spending less time outdoors and participating in physical activity, and more time gaming or using social media (Sciberras et al., 2022; Shah et al, 2021). These factors could also be considered by parents and clinicians when addressing stress in children with ADHD. Other considerations may include diet and sleep (Behrmann et al., 2022). Parents may require support to manage their own stress to be able to help their children cope during the pandemic and address any adverse impacts on ADHD symptoms, particularly hyperactivity/impulsivity. A family systems approach may be helpful to improve coping strategies and mental health (Cortese et al., 2020; Organisation for Economic Cooperation and Development, 2021). This approach is appropriate to be delivered via telehealth to support families of children with ADHD in the case of isolation or other social distancing restrictions (McKee et al., 2022).

The results of this study should be interpreted in the context of some limitations. First, we only examined baseline levels of COVID-19 stress. It is unknown how COVID-19 stress changed over the 12-month period given that many parts of Australia experienced additional lockdowns and restrictions after the baseline survey. Furthermore, parent reports may be influenced by their own stress and worries due to the pandemic. Future research should consider gaining children’s perspectives during the pandemic to assess any discrepancies between parent and child reports. Moreover, no control group was used in the study, so it is unknown whether these results are specific to children with ADHD. However, based on previous findings (Breaux et al., 2021), it is likely that the associations shown in the study would be more pronounced in children with ADHD compared to their non-ADHD peers. It should also be noted that co-occurring internalizing conditions were higher in the sample than expected.

The study also had multiple strengths. First, the retention of participants was relatively high across the 12 months. Second, the study controlled for additional factors such as ADHD medication use, socio-economic indicators, and baseline symptoms. This allowed the impact of COVID-19 stress to be assessed independently from other factors known to influence ADHD, oppositional, and mental health symptoms. This addressed a common limitation of previous studies focusing on ADHD during the pandemic (Melegari et al., 2021; Shah et al., 2021; Zhang et al., 2020).

Since the 12-month survey, multiple parts of Australia have experienced additional lockdowns and a return to remote learning. New South Wales and Victoria experienced a rise in case numbers in July and August 2021, prompting extended lockdowns in these states. During this time, Melbourne, Victoria became the most locked-down city in the world (Boaz, 2021). The number of cases of COVID-19 in Australian children has also risen compared to 2020 (Cockburn, 2021), which may contribute to increased COVID-19 stress in children. Future research could also expand upon the current study by considering any differences in COVID-19 stress for primary-school aged children versus high school students, or between states of Australia. Given the small but significant association identified between COVID-19 stress and ADHD symptoms, it is important for research to continue to monitor any longer-term outcomes.

In conclusion, baseline COVID-19 stress was associated with increased ADHD symptom severity 12 months later, specifically hyperactivity/impulsivity symptoms, in children aged 5 to 17 years with ADHD. These long-term associations were independent of baseline symptoms and other demographic factors, co-occurring conditions, and ADHD medication use. Children with ADHD and their families may require additional support to cope with ongoing COVID-19 stress as the pandemic continues. Future research should continue to investigate the longer-term impacts of the COVID-19 pandemic, and the influence of stress on the mental health of children with ADHD over time.

## Supplemental Material

sj-docx-1-jad-10.1177_10870547231168334 – Supplemental material for Longitudinal Associations Between COVID-19 Stress and the Mental Health of Children With ADHD Click here for additional data file. Supplemental material, sj-docx-1-jad-10.1177_10870547231168334 for Longitudinal Associations Between COVID-19 Stress and the Mental Health of Children With ADHD by Ainsley Summerton, Susannah T. Bellows, Elizabeth M. Westrupp, Mark A. Stokes, David Coghill, Mark A. Bellgrove, Delyse Hutchinson, Stephen P. Becker, Glenn Melvin, Jon Quach, Daryl Efron, Argyris Stringaris, Christel M. Middeldorp, Tobias Banaschewski and Emma Sciberras in Journal of Attention Disorders
