# Supplementary material for: Longitudinal Associations Between COVID-19 Stress and the Mental Health of Children With ADHD
Source: J Atten Disord. 2023 Apr 25;27(10):1065–74. doi: 10.1177/10870547231168334 (PMC10130929; doi:10.1177/10870547231168334)
Supplement: sj-docx-1-jad-10.1177_10870547231168334 – Supplemental material for Longitudinal Associations Between COVID-19 Stress and the Mental Health of Children With ADHD [file sj-docx-1-jad-10.1177_10870547231168334.docx]

**Supplementary Materials**

Figure 1. *Flowchart of Participants Across 12 Months*

Table 1. *Means, Standard Deviations and Range for Independent and Dependent Variables*

Table 2. *Bivariate Correlations Between Variables of Interest*

Table 3. *Association Between Baseline COVID-19 Stress and 12 Month Functioning Controlling for Covariates and Baseline Symptoms*

Supplementary Figure 1.

*Flowchart of Participants Across 12 Months*

Lost to follow-up (*n* = 6)

## Baseline survey

## 12-month follow-up

Consent given (*n* = 221)

Did not meet inclusion criteria (*n* = 8)

## Recruitment

Baseline survey completed (*n* = 213)

Insufficient data for analysis (*n* = 5)

Lost to follow-up (*n* = 4)

Incomplete (*n* = 4)

Did not respond (*n* = 56)

## Analysis

Eligible for analysis (*n* = 140)

Follow-up survey completed (*n* = 145)

Follow-up survey invitations (*n* = 207)

Supplementary Table 1.
*Means, Standard Deviations and Range for Independent and Dependent Variables*

|  | **Baseline**^a^ | |  | **12-months**^b^ | |
| --- | --- | --- | --- | --- | --- |
| **Measure** | *M* (*SD*) | Range |  | *M* (*SD*) | Range |
| COVID-19 related stress | 2.6 (0.9) | 1.0 – 5.0 |  | – | – |
| ADHD symptom severity  Inattention  Hyperactivity/impulsivity | 33.1 (11.5)  18.7 (6.0)  14.4 (7.0) | 5.0 – 54.0  3.0 – 27.0  1.0 – 27.0 |  | 30.1 (9.8)  17.1 (5.2)  13.0 (5.9) | 11.4 – 52.0  4.0 – 27.0  0.0 – 27.0 |
| Opposition | 11.7 (6.6) | 0.0 – 24.0 |  | 11.1 (5.4) | 0.0 – 24.0 |
| Negative affect | 3.2 (0.7) | 1.3 – 4.9 |  | 2.7 (0.6) | 1.6 – 4.6 |
| Anxiety | 10.0 (5.1) | 0.0 – 24.0 |  | 9.4 (4.8) | 0.0 – 24.0 |
| Depression | 9.6 (5.7) | 1.0 – 26.0 |  | 8.7 (5.7) | 0.0 – 23.0 |
| Irritability | 5.4 (3.5) | 0.0 – 12.0 |  | 4.9 (3.3) | 0 – 12.0 |

*Note.* ^a^ *n* = 120–140 ^b^ *n* = 138–140

Supplementary Table 2.

*Bivariate Correlations Between Variables of Interest*

| Variable | 1. | 2. | 3. | 4. | 5. | 6. | 7. | 8. | 9. |
| --- | --- | --- | --- | --- | --- | --- | --- | --- | --- |
| 1. Baseline COVID-19 related stress | – |  |  |  |  |  |  |  |  |
| 2. 12-month ADHD symptom severity | .221** | – |  |  |  |  |  |  |  |
| 3. 12-month inattention | .136 | .862** | – |  |  |  |  |  |  |
| 4. 12-month hyperactivity/impulsivity | .247** | .897** | .550** | – |  |  |  |  |  |
| 5. 12-month opposition | .185* | .592** | .478** | .558** | – |  | ­ |  |  |
| 6. 12-month anxiety | .101 | .243** | .254** | .180* | .278** | – |  |  |  |
| 7. 12-month depression | .084 | .334** | .382** | .218** | .434** | .594** | – |  |  |
| 8. 12-month irritability | .142 | .460** | .402** | .408** | .771** | .305** | .518** | – |  |
| 9. 12-month negative affect | .198* | .391** | .405** | .290** | .472** | .591** | .693** | .560** | – |
| 10. Baseline ADHD symptom severity | .098 | .547** | .436** | .533** | .382** | .150 | .255** | .290** | .238** |
| 11. Baseline inattention | .110 | .411** | .449** | .293** | .269** | .144 | .260** | .207* | .259** |
| 12. Baseline hyperactivity/impulsivity | .068 | .551** | .336** | .629** | .400** | .125 | .198* | .302** | .172 |
| 13. Baseline opposition | .198* | .368** | .306** | .348** | .581** | .145 | .290** | .490** | .224* |
| 14. Baseline anxiety | .043 | .131 | .138 | .098 | .179 | .530** | .307** | .148 | .252** |
| 15. Baseline depression | .336** | .206* | .251** | .124 | .302** | .339** | .461** | .283** | .308** |
| 16. Baseline irritability | .265** | .302** | .235** | .300** | .525** | .172 | .247** | .504** | .262** |
| 17. Baseline negative affect | .584** | .291** | .245** | .269** | .364** | .314** | .314** | .358** | .418** |
| 18. ADHD medication use | -.012 | -.172* | -.132 | -.169* | -.104 | -.052 | -.008 | -.015 | -.072 |
| 19. Child age | .073 | -.281** | -.104 | -.373** | -.088 | .140 | .136 | -.003 | .107 |
| 20. Child gender | -.014 | .026 | .006 | .037 | .085 | -.043 | -.010 | -.059 | .037 |
| 21. SEIFA^a^ | .082 | .012 | .083 | -.052 | -.044 | .133 | .096 | -.019 | .071 |

*Note.* *N* = 116–140 * *p* < .05, ** *p* < .01 ^a^ Socio-Economic Indexes for Areas (SEIFA)

Supplementary Table 3.

*Association Between Baseline COVID-19 Stress and 12 Month Functioning Controlling for Covariates and Baseline Functioning*

|  | R^2^ | *Adj.* R^2^ | *p* | *B* [95% CI] | β | *p* |
| --- | --- | --- | --- | --- | --- | --- |
| **12-month ADHD symptom severity** | .37 | .34 | <.001 |  |  |  |
| Baseline COVID-19 related stress |  |  |  | 2.43 [0.67, 4.19] | 0.21 | .007 |
| Baseline ADHD symptom severity |  |  |  | 0.40 [0.27, 0.54] | 0.46 | <.001 |
| ADHD medication use |  |  |  | -4.18 [-9.68, 1.32] | -0.12 | .135 |
| Child age |  |  |  | -0.61 [-1.13, -0.09] | -0.19 | .022 |
| Child gender |  |  |  | -1.53 [-4.98, 1.92] | -0.68 | .382 |
| SEIFA^a^ |  |  |  | 0.01 [-0.02, 0.04] | 0.07 | .376 |
| **12-month inattention** | .24 | .20 | <.001 |  |  |  |
| Baseline COVID-19 related stress |  |  |  | 0.82 [-0.20, 1.84] | 0.13 | .113 |
| Baseline inattention |  |  |  | 0.37 [0.22, 0.52] | 0.42 | <.001 |
| ADHD medication use |  |  |  | -1.91 [-5.10, 1.28] | -0.10 | .238 |
| Child age |  |  |  | -0.16 [-0.45, 0.13] | -0.10 | .264 |
| Child gender |  |  |  | -0.63 [-2.63, 1.37] | -0.05 | .533 |
| SEIFA |  |  |  | 0.01 [-0.01, 0.03] | 0.08 | .325 |
| **12-month hyperactivity/impulsivity** | .47 | .44 | <.001 |  |  |  |
| Baseline COVID-19 related stress |  |  |  | 1.59 [0.62, 2.55] | 0.23 | .002 |
| Baseline hyperactivity/impulsivity |  |  |  | 0.45 [0.32, 0.58] | 0.52 | <.001 |
| ADHD medication use |  |  |  | -2.25 [-5.27, 0.77] | -0.10 | .142 |
| Child age |  |  |  | -0.41 [-0.70, -0.11] | -0.21 | .008 |
| Child gender |  |  |  | -0.93 [-2.83, 0.96] | -0.07 | .332 |
| SEIFA |  |  |  | 0.01 [-0.01, 0.02] | 0.05 | .517 |
| **12-month opposition** | .36 | .32 | <.001 |  |  |  |
| Baseline COVID-19 related stress |  |  |  | 0.67 [-0.34, 1.68] | 0.10 | .191 |
| Baseline opposition |  |  |  | 0.47 [0.33, 0.61] | 0.56 | <.001 |
| ADHD medication use |  |  |  | -2.02 [-5.07, 1.03] | -0.10 | .191 |
| Child age |  |  |  | -0.13 [-0.41, 0.15] | -0.07 | .356 |
| Child gender |  |  |  | 0.04 [-1.91, 1.98] | 0.003 | .971 |
| SEIFA |  |  |  | 0.01 [-0.01, 0.02] | 0.07 | .347 |
| **12-month negative affect** | .24 | .20 | <.001 |  |  |  |
| Baseline COVID-19 related stress |  |  |  | -0.05 [-0.19, 0.09] | -0.07 | .468 |
| Baseline negative affect |  |  |  | 0.40 [0.24, 0.56] | 0.50 | <.001 |
| ADHD medication use |  |  |  | -0.17 [-0.50, 0.17] | -0.08 | .328 |
| Child age |  |  |  | 0.01 [-0.02, 0.05] | 0.07 | .431 |
| Child gender |  |  |  | 0.11 [-0.12, 0.35] | 0.08 | .335 |
| SEIFA |  |  |  | 0.001 [-0.001, 0.003] | 0.12 | .203 |
| **12-month anxiety** | .33 | .29 | <.001 |  |  |  |
| Baseline COVID-19 related stress |  |  |  | 0.16 [-0.74, 1.06] | 0.03 | .722 |
| Baseline anxiety |  |  |  | 0.52 [0.37, 0.67] | 0.54 | <.001 |
| ADHD medication use |  |  |  | -1.73 [-4.50, 1.05] | -0.99 | .220 |
| Child age |  |  |  | 0.15 [-0.11, 0.41] | 0.09 | .252 |
| Child gender |  |  |  | 0.05 [-1.72, 1.82] | 0.004 | .956 |
| SEIFA |  |  |  | 0.01 [-0.01, 0.02] | 0.10 | .231 |
| **12-month depression** | .25 | .21 | <.001 |  |  |  |
| Baseline COVID-19 related stress |  |  |  | -0.35 [-1.51, 0.80] | -0.05 | .547 |
| Baseline depression |  |  |  | 0.49 [0.31, 0.66] | 0.49 | <.001 |
| ADHD medication use |  |  |  | -0.77 [-4.16, 2.62] | -0.04 | .655 |
| Child age |  |  |  | 0.11 [-0.21, 0.42] | 0.06 | .505 |
| Child gender |  |  |  | -0.41 [-2.58, 1.76] | -0.03 | .708 |
| SEIFA |  |  |  | 0.01 [-0.01, 0.03] | 0.08 | .383 |
| **12-month irritability** | .28 | .24 | <.001 |  |  |  |
| Baseline COVID-19 related stress |  |  |  | 0.12 [-0.56, 0.78] | 0.03 | .745 |
| Baseline irritability |  |  |  | 0.52 [0.35, 0.68] | 0.53 | <.001 |
| ADHD medication use |  |  |  | -0.73 [-2.72, 1.27] | -0.06 | .471 |
| Child age |  |  |  | 0.05 [-0.14, 0.23] | 0.04 | .609 |
| Child gender |  |  |  | -0.76 [-2.03, 0.52] | -0.10 | .243 |
| SEIFA |  |  |  | 0.003 [-0.01, 0.01] | 0.05 | .591 |

*Note. N* = 116–119 ^a^ Socio-Economic Indexes for Areas (SEIFA)
